# Long-term application of silver nanoparticles in dental restoration materials: potential toxic injury to the CNS

**DOI:** 10.1007/s10856-023-06753-z

**Published:** 2023-10-19

**Authors:** Kaimei Wang, Shiqi Wang, Jingju Yin, Qiankun Yang, Yi Yu, Lin Chen

**Affiliations:** 1 Guiyang Hospital of Stomatology, Guiyang, Guizhou Province 563000 China; 2 The Medical unit of 65651 troops of Chinese people’s Liberation Army, Jinzhou, Liaoning Province 121100 China; 3 Fujian Medical University; Department of Stomatology, The First Affiliated Hospital of Fujian Medical University, Fuzhou, Fujian Province 350002 China; 4 The Southwest Hospital of Army Medical University, Chongqing, 400038 China; 5 Hospital of Stomatology, Zunyi Medical University, Zunyi, Guizhou Province 563100 China

## Abstract

**Abstract:**

Silver nanoparticles (AgNPs) have durable and remarkable antimicrobial effects on pathogenic microorganisms, such as bacteria and fungi, in dental plaques. As such, they are widely added to dental restoration materials, including composite resins, denture bases, adhesives, and implants, to solve the problems of denture stomatitis, peri-implant inflammation, and oral infection caused by the long-term use of these dental restoration materials. However, AgNPs can be absorbed into the blood circulatory system through the nasal/oral mucosa, lungs, gastrointestinal tract, skin, and other pathways and then distributed into the lungs, kidneys, liver, spleen, and testes, thereby causing toxic injury to these tissues and organs. It can even be transported across the blood-brain barrier (BBB) and continuously accumulate in brain tissues, causing injury and dysfunction of neurons and glial cells; consequently, neurotoxicity occurs. Other nanomaterials with antibacterial or remineralization properties are added to dental restoration materials with AgNPs. However, studies have yet to reveal the neurotoxicity caused by dental restoration materials containing AgNPs. In this review, we summarize the application of AgNPs in dental restoration materials, the mechanism of AgNPs in cytotoxicity and toxic injury to the BBB, and the related research on the accumulation of AgNPs to cause changes of neurotoxicity. We also discuss the mechanisms of neurotoxicity caused by AgNPs and the mode and rate of AgNPs released from dental restorative materials added with AgNPs to evaluate the probability of neurotoxic injury to the central nervous system (CNS), and then provide a theoretical basis for developing new composite dental restoration materials.

**Graphical Abstract:**

**Figure:**
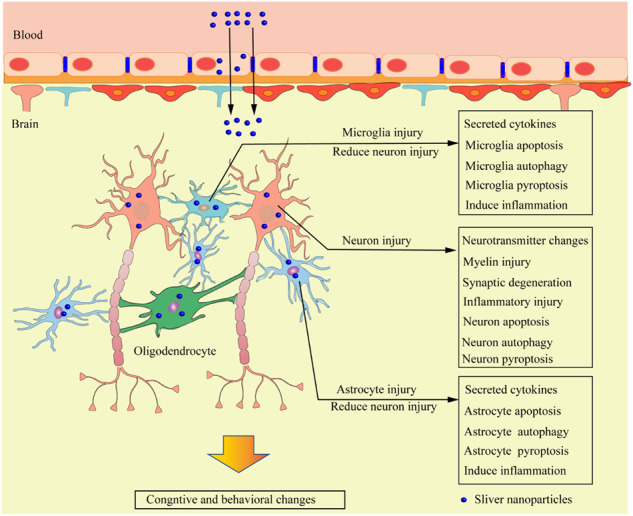
**Mechanism of neurotoxicity caused by AgNPs**: AgNPs in the blood circulation enter the brain tissue after being transported across the BBB through transendothelial cell pathway and paracellular transport pathway, and continuously accumulate in brain tissue, causing damage and dysfunction of neurons and glial cells which ultimately leads to neurotoxicity. The uptake of AgNPs by neurons, astrocytes and microglia causes damage to these cells. AgNPs with non-neurotoxic level often increases the secretion of a variety of cytokines, up-regulates the expression of metallothionein in glial cells, even up-regulates autophagy and inflammation response to protect neurons from the toxic damage of AgNPs. However, the protective effect of glial cells induced by AgNPs exposure to neurotoxic levels is insufficient, which leads to neuronal damage and dysfunction and even neuronal programmed cell death, eventually cause neurotoxicity.

## Introduction

A dental plaque biofilm is a colony composed of Gram-positive bacteria, Gram-negative bacteria, anaerobic bacteria, fungi, and other microorganisms on the surface of teeth. Most of the time, these microorganisms are in a stable state. However, once the homeostasis of biofilms is disrupted, dental caries, periodontitis, and other oral diseases occur [1, 2]. Dental plaque biofilms can also be retained on the surface of filling materials, denture bases, or implant materials used in the treatment of patients with dental caries or dentition defects. The imbalance of dental plaque biofilms on the surface of dental restorative materials can lead to secondary caries, denture stomatitis, implant infection, and oral infection, eventually resulting in the failure of prosthodontic therapy. These biofilms can also effectively protect internal pathogenic microorganisms from the immune system of host organisms; as such, they cannot be completely eliminated and killed by daily-use oral detergents and antibiotic drugs [3, 4]. To prevent and treat the formation of dental plaque biofilms, researchers applied nanotechnology to develop new methods.

Before the discovery of antibiotics, the most commonly used antibacterial agent was silver. AgNPs have been prepared through physical, chemical, and biological synthesis methods. They have a diameter of 1–100 nm, an ultrasmall particle size, a large specific surface area, high charge, and other excellent physical properties. They can effectively interact with pathogenic bacteria, penetrate the membrane and wall of bacteria, enter bacterial cells, and release Ag^+^ to produce an antibacterial effect. Although the antibacterial mechanism of AgNPs has been widely studied, it has not been explained clearly. The antibacterial properties of AgNPs mainly depend on the particle size, shape, concentration, surface properties, and aggregation state of AgNPs. In particular, AgNPs with a small particle size and a large specific surface area more easily penetrate the membrane and wall of bacterial cells, so their antibacterial effects were improved [5]. Compared to other nanoparticles, AgNPs were more sensitive to the level of oxygen and they can effectively catalyze the conversion of oxygen into reactive oxygen species (ROS) and produce better antibacterial effect. One cann’t imagine they also have excellent antibacterial effects on multidrug-resistant pathogenic bacteria. Therefore, AgNPs have become the preferred additive agent for developing new composite dental restoration materials with highly antibacterial effects and physical and mechanical properties. In addition, the addition of AgNPs in denture base materials can enhance the antiaging properties and exceptional bio-compatibility [6], and the modification of AgNPs on the surface of the implant can also enhance implant osseointegration through improving the surrounding microenvironment of the dental implants [7]. However, the unique physical and chemical properties of AgNPs have limitations; that is, their widespread use may cause toxic reactions in organisms. Therefore, studies should explore this problem and provide solutions. Studies have confirmed that AgNPs can be absorbed into the blood circulatory system through various pathways, such as the nasal/oral mucosa, lungs, gastrointestinal tract, and skin, and distributed to the lungs, kidneys, liver, spleen, testes, and other tissues and organs, eventually causing toxic injury to those tissues and organs [8, 9]. Significantly, some studies have even found that AgNPs can be transported across the BBB and enter the brain tissue. Unlike tissues or organs such as the liver and kidneys, the brain can be penetrated by AgNPs and release Ag^+^, which is difficult to eliminate, so these ions accumulate continuously. Therefore, low-dose AgNPs can induce neurotoxicity through slow accumulation [8, 10].

Although AgNPs have been commonly added to dental restorative materials, few studies have been performed on toxic injuries of these materials to the CNS. In this review, we discussed the application of AgNPs in dental restorative materials, the mechanism of AgNPs in cytotoxicity and impairment of BBB, and the progression of the accumulation of AgNPs to cause neurotoxicity in CNS. Then, the probability of neurotoxicity caused by dental restorative materials added with AgNPs was evaluated.

## Application of AgNPs in dental restorative materials

Dental caries and periodontal disease are common oral diseases. With disease progression, the affected teeth lose vitality and even gradually loosen and fall off. In clinical treatment, most patients with dental caries receive composite resin materials for restoration. For teeth that cannot be filled, full crowns and inlays can be used to retain the natural teeth as much as possible, but teeth with serious defects, especially those subjected to poorly effective root canal treatment should be extracted. For patients with dentition defects caused by tooth extraction or periodontitis, removable or implant dentures can be used to restore their masticatory function and facial appearance. Implant dentures can effectively retain the alveolar bone tissue of missing teeth and the normal position of the teeth adjacent to the missing teeth. They also improve patients’ masticatory function and quality of life, and the facial esthetic effect is prominent. Furthermore, the success rate of implant restoration treatment is more than 95%, so patients with dentition defects gradually abandon traditional denture restoration and choose implant denture restoration [11]. By 2026, 23% of adult edentulous patients in the United States will have chosen implant denture; however, removable denture is a cheaper and still the preferred treatment for many patients affected by economic factors [12]. Patients with tooth defects, dentition defects, and dentition loss are treated with composite resin, ceramics, implant materials, and adhesives, but these materials are constantly aging because of saliva, chewing, brushing, and other factors in the oral cavity. Their surfaces are also vulnerable to the invasion of bacteria in the oral cavity microenvironment and adherence of restoration materials to the surface to form a biofilm. Once the biofilm is formed, removing it completely via conventional cleaning methods, such as antibiotics and brushing teeth, is difficult; as such, biofilm formation mainly causes secondary caries, caries demineralization and rupture or even fracture, ceramic restoration rupture, denture stomatitis, and peri-implantitis, which finally lead to the failure of restoration treatment [3, 13]. Studies have shown that about 17.8 different types of bacteria grow on the surface of the test material after polymethyl methacrylate (PMMA) is placed in the oral cavity for 1 month [14], and the 5-year failure rate of patients after adhesive repair treatment is as high as 50% [15]. To solve the defects of traditional dental restorative materials, such as lack of antibacterial properties and insufficient physical and mechanical properties, researchers developed composite restorative materials with antibacterial properties by doping AgNPs into composite resins, adhesives, and ceramics and loading them on the surface of implant materials; they found that some physical and mechanical properties of these materials have been improved [16–18]. The concentration of AgNPs can significantly affect the antibacterial and physicochemical properties of dental restoration materials; that is, the higher the concentration of AgNPs is, the better the antibacterial effect of dental restoration materials will be. However, a further increase in the concentration of AgNPs causes the physicochemical properties of dental restoration materials to decrease and leads to poor biological safety. In vitro studies have demonstrated that <10 μg/ml AgNPs are noncytotoxic to osteoblasts and can induce osteoblast proliferation and cytokine release, but it has poor antibacterial effects [19]. Therefore, AgNPs should be incorporated at an appropriate concentration. In addition to AgNPs as dental restoration materials, nanomaterials, including antibacterial agents (e.g., Quaternary ammonium dimethacrylate (QADM) and graphene), metal oxides (e.g., TiO_2_, SiO_2_, ZnO, and ZrO_2_), or remineralization agents (e.g., Amorphouscalcium phosphate (NACP), Hydroxyapatite (HA), and CaP), together with AgNPs, are included in dental restorative materials to enhance their antibacterial performance and remineralization performance. For these reasons, they have emerged as the main components for the development and preparation of new-generation multifunctional composite dental restorative materials with durable antibacterial effect, remineralization performance, excellent physicochemical properties, and biological safety [20, 21] Table 1.

**Table 1:** Summary of the application of AgNPs in dental restoration materials

Application	Types of additive agents	Influence of dental restoration materials	Ref.
Composite resin	AgNPs	The viscosity of the composite resin increases exponentially as the concentration of AgNPs increases, and the conversion rate decreases continuously. The bending strength, elastic modulus, and impact strength of composite resin initially increase and then decrease with the concentration of AgNPs. The water absorption of composite resin is not significantly affected by AgNPs addition.	[141]
	TiO_2_, TiO_2_/AgNPs	The increase in the content of AgNPs added to the composite resin significantly reduced bacterial growth, and the surface roughness increases as the concentration of nanoparticles increases. The addition of 2% TiO_2_/AgNPs significantly reduces the biofilm accumulation of *Streptococcus mutans* on the surface of the composite resin compared with that of the control group.	[142]
	ZnO, ZnO/AgNPs	ZnO/AgNPs form synergistic antibacterial properties to enhance antibacterial effects and have no significant influence on the mechanical properties. Ag^+^ released by AgNPs and Zn^2+^ released by nano-ZnO can synergistically induce the generation and accumulation of ROS in bacteria, eliciting a superimposed effect on the antibacterial ability of Gram-positive and Gram-negative bacteria.	[143]
	AgNPs–NACP	The mechanical properties of the composite resin added with 0–0.042% NACP are equivalent to those of commercial composites without antibacterial activity and have no adverse effect on the bearing performance of the composites against the intraoral chewing force. AgNPs–NACP have excellent antibacterial properties, significantly reducing biofilm activity and lactic acid production.	[144]
	Ciprofloxacin (CIP) –AgNPs	The addition of CIP-AgNPs to composite resin has enabled the materials to exhibit antimicrobial activity against *Streptococcus mutans, Streptococcus sobrinus*, and the salivary microbiota. Compared to the control group, the composite resin material containing CIP-AgNPs has increased antibacterial activity and compressive strength, as well as excellent biocompatibility.	[145]
Ceramics	AgNPs, Titanium (Ti), AgNPs-Ti	Ti or AgNPs can significantly increase the fracture toughness of feldspathic ceramics, and the addition of high-concentration AgNPs–Ti reduces the fracture resistance of feldspathic ceramics.	[146]
	AgNPs	As the concentration of AgNPs in dental ceramics increases, the stress corrosion susceptibility coefficient characterizing the subcritical crack growth behavior increases. AgNPs addition can effectively inhibit the fatigue fracture of dental ceramics.	[147]
	AgNPs, Platinum nanoparticles	The mechanical properties of ceramics are enhanced by adding AgNPs or platinum nanoparticles. The addition of AgNPs and platinum nanoparticles improves Young’s modulus and fracture toughness compared with a NS porcelain without metal nanoparticles. The effect of AgNPs on improving fracture toughness is stronger than that of platinum nanoparticles.	[148]
PMMA	AgNPs	After AgNPs are added into PMMA, the material can produce an excellent antibacterial effect by releasing AgNPs and Ag^+^, reduce surface roughness, and effectively reduce the adhesion and colonization of biofilms, especially in the case of a high addition ratio. The excessive addition ratio of AgNPs results in limited physicochemical properties of the material.	[149–152]
	Nano metal oxides (e.g., TiO_2_, SiO_2_, ZnO and ZrO_2_)	Metal oxide nanoparticles improve the physicochemical properties of PMMA, but the use of these additives is limited because of their poor antibacterial effect and potential cytotoxicity.	[153, 154]
	Nanosliver-loaded inorganic antibacterial agents (Nanosliver-loaded TiO_2_/SiO_2_/ZnO/ZrO_2_)	Adding Nanosliver-loaded inorganic antibacterial agents into PMMA can change the release mode and pattern of Ag^+^. After PMMA is added, the antibacterial and physicochemical properties of the material are improved, and the easy accumulation of AgNPs in PMMA is avoided.	[155, 156]
	Graphene nanoparticles	PMMA doped with graphene nanoparticles has excellent mechanical properties and antibacterial properties.	[157, 158]
	Graphene -AgNPs	Graphene-AgNPs added to PMMA at 1% and 2% mass percentage produce materials with improved mechanical properties, such as compressive behavior, flexural strength, and tensile strength. When the mass percentage is 1%, the fracture modulus of the material increases by 174%, which further shows that the addition of graphene–Ag nanoparticles can yield PMMA with high flexibility and toughness.	[159]
	Graphene-AgNPs	The material added with graphene-AgNPs, have an excellent bactericidal effect on *Staphylococcus aureus, Streptococcus mutans*, and *Escherichia coli*. It has no significant effect on the cell viability of dysplastic oral keratinocytes and dental pulp stem cells.	[160]
Ti implant	AgNPs	AgNPs loaded on the surface of Ti have an excellent antibacterial effect on various microorganisms. The higher the concentration of loaded AgNPs on the surface of Ti is, the better the antibacterial effect of Ti will be. However, when the concentration of loaded AgNPs increases, the surface roughness of Ti increases, whereas the wettability decreases. The material has an obvious cytotoxic effect on osteoblasts.	[131, 161]
	Nano-porous silica (NSC), AgNPs/NSC	NSC loaded on the surface of Ti effectively improves the osseointegration characteristics of Ti; AgNPs/NSC loaded on the surface of Ti reduces the survival rate of bacteria, and the biofilm coverage by more than 50% compared with the unloaded ones. It also decreases bacterial adhesion.	[162, 163]
	AgNPs-Polydopamine (PDA)	AgNPs–PDA loaded on the surface of Ti have excellent antibacterial effects against *Streptococcus mutans* and Porphyromonas gingivalis.	[164]
	Double-layer TiO2 nanotubes (DNT) -AgNPs	In vitro studies have shown that the release rate of Ag+ from DNT-AgNPs coated implant materials is slow, with an antimicrobial efficiency of 55.6%. Furthermore, it has been demonstrated that these coated materials do not negatively affect the adhesion, viability, proliferation, ALP staining, or activity of rat bone marrow mesenchymal stem cells. On the contrary, they increase the expression of osteogenic genes in these cells. In vivo studies have also shown that the DNT-AgNPs coated implant materials promote bone-implant osseointegration in a beagle mandibular tooth loss model.	[165]
	TiO_2_ nanotubes-PDA-AgNPs	TiO2 nanotubes-PDA-AgNPs coating is formed on the surface of Ti. Coatings can strongly resist oxidation and ROS-scavenging bioactivity. The composite antibacterial coating changes the release kinetics of the AgNPs the material from a sudden release mode to the controlled release mode. The release rate of Ag^+^ is slower than that of AgNPs-PDA coating and PDA-TiO_2_ coating and has a better and longer bactericidal effect on methicillin-resistant *Staphylococcus aureus*. It also promotes the adhesion, proliferation, and spreading of preosteoblast bone cells, which effectively improve the osseointegration ability of Ti.	[135]
Dental adhesives	AgNPs	After AgNPs are added to the dental adhesive, the adhesive properties of the material are not affected. They can also kill residual bacteria in the tooth cavity and the bacteria that invade and colonize the edge of the restoration even if dental adhesive is added with a low dose of AgNPs, it also has a good antibiofilm effect while maintaining the bonding strength and biocompatibility of the adhesive.	[166]
	AgNPs-QADM	The antibacterial ability of the adhesive added with AgNPs–QADM is stronger than that of the adhesive with AgNPs alone. The former also does not affect the adhesive force of dentin.	[167]
	AgNPs-NACP	The addition of AgNPs–NACP to adhesives greatly reduces the vitality of biofilm and acid production but does not reduce the adhesive force of adhesives.	[168]
	AgNPs-NACP-Glass ionomer cements (GIC); AgNPs–HA-GIC	Both AgNPs–NACP-GIC and AgNPs–HA-GIC have better antibacterial properties. The concentration of Ag^+^ were released by GIC after the addition of nanocomposite antibacterial agents is lower than the detection limit (1 mg/ml). It does not affect the compressive strength of GIC without changing the release of fluoride ions.	[136]

## Cell uptake of AgNPs

**Fig. 1:**
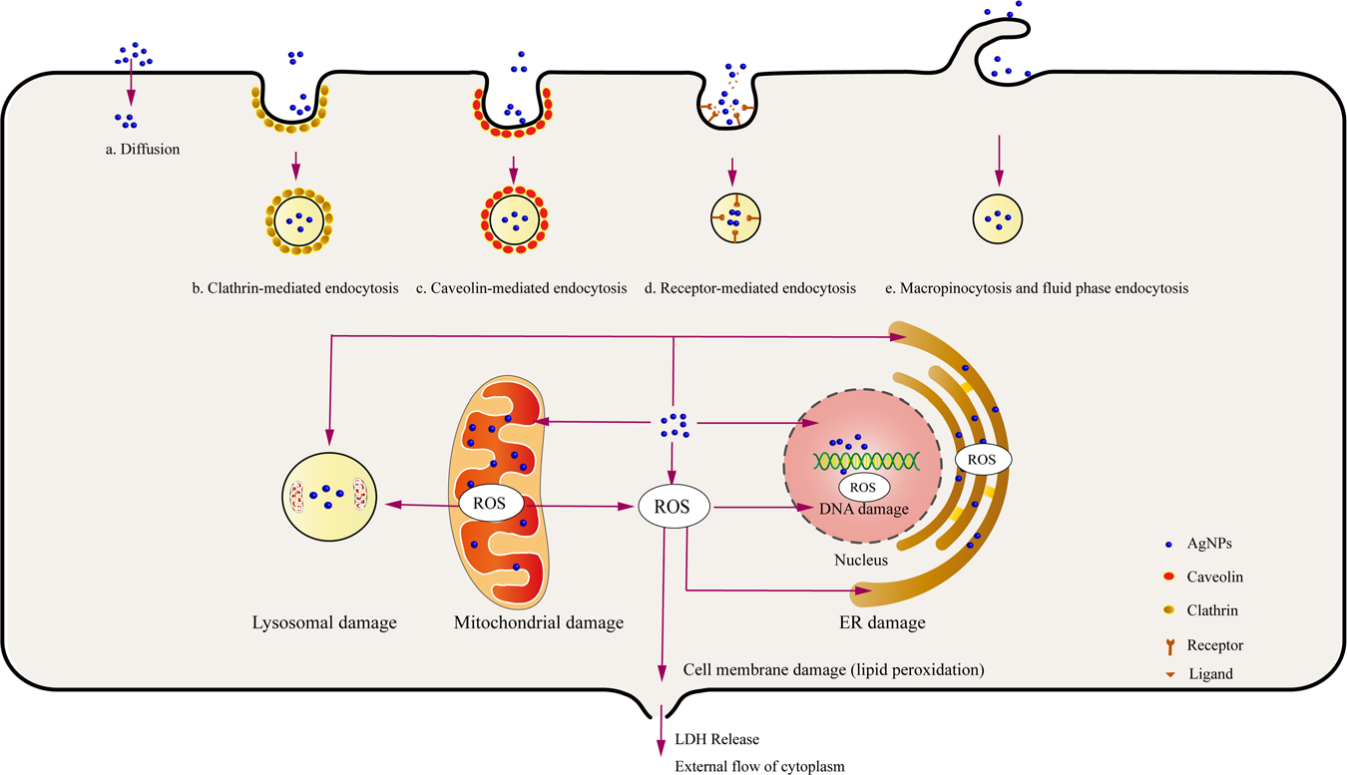
Pathways of cellular uptake of AgNPs. (1) AgNPs with smaller particle size: **a** diffusion. (2) AgNPs with a large particle size: **b** clathrin-mediated endocytosis, **c** caveolin-mediated endocytosis, **d** receptor-mediated endocytosis, and **e** macropinocytosis and fluid phase endocytosis. AgNPs ingest cells; deposit organelles such as the mitochondria, ER, and nucleus; and generate ROS, which cause damage and dysfunction of these organelles and lipid peroxidation of the cell membrane, resulting in the damage and even rupture of the cell membrane and eventually leading to LDH release and external flow of the cytoplasm

After coming in contact with the cell membrane, AgNPs degrade or become eliminated through cell membrane receptor recognition, internalization, and translocation. AgNPs with different sizes are absorbed by cells in different pathways. For example, AgNPs with a small particle size (about 10 nm) can directly penetrate the cell membrane or enter cells through endocytosis to release Ag^+^ and interact with various biomolecules in cells. Conversely, AgNPs with a large particle size (more than 100 nm) cannot directly penetrate the cell membrane, so they undergo endocytosis (clathrin-mediated endocytosis, receptor-mediated endocytosis, macropinocytosis, and fluid-phase endocytosis) to enter cells. During endocytosis, AgNPs become absorbed into early endosomes formed by the cell membrane and gradually develop into late endosomes; finally, they form lysosomes with low pH. Lysosomes in an acidic environment can increase the release of Ag^+^ from AgNPs and promote toxic injury to cells [8, 22]. ROS production induced by AgNPs leads to lipid oxidation of the cell membrane, resulting in abnormal membrane function, increased permeability, and even membrane rupture, which is more conducive to the occurrence of toxic reactions triggered by AgNPs entering cells or organelles [23]. These AgNPs that enter the cells continue to be deposited in organelles such as the mitochondria, Endoplasmic reticulum (ER), and lysosomes, causing cellular oxidative stress; some AgNPs can pass through the nuclear membrane and enter the nucleus, causing DNA damage. Consequently, these changes can lead to cell cycle arrest, inflammation, and programmed cell death (Fig. 1) [24].

## Mechanism of cytotoxicity mediated by AgNPs

**Fig. 2:**
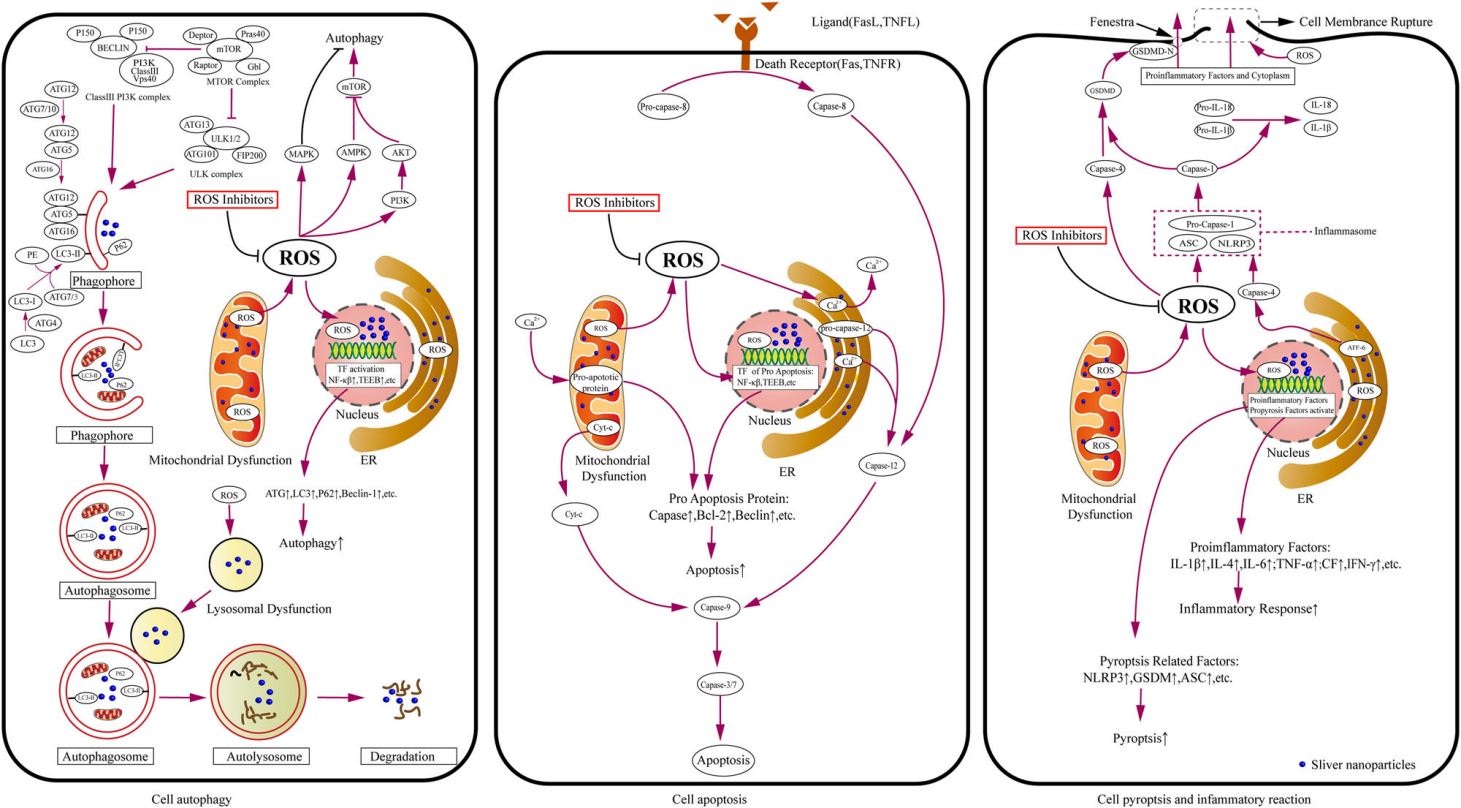
Mechanism of cytotoxicity induced by AgNPs. (1) In autophagy, AgNPs induce cellular autophagy by inducing the production of intracellular ROS and then activating signaling pathways, including PI3K-AKT-mTOR, AMPK-mTOR and MAPK (JNK, p38, and ERK). (2) In apoptosis, AgNPs induce cellular autophagy by inducing the production of intracellular ROS and then activating apoptotic pathways, including mitochondrial pathway, death receptor pathway, and ER stress pathway. (3) In pyroptosis and inflammatory response, AgNPs induce cellular oxidative stress, mitochondrial damage, ER stress, and lysosomal disorder to further activate the inflammatory body complex composed of NLRP-3, ASC, and pro-Caspase-1. This complex then mediates Caspase-1 activation that promotes the formation and release of IL-1β and IL-18 to mediate inflammatory responses. The activated Caspase-1 also cleaves GSDM to allow the N-terminal domain of GSDMD to form pores in the plasma membrane and eventually cause cell pyroptosis and inflammatory response. (4) AgNPs can activate transcription factors, such as NF-κB and TFEB; upregulate the expression of cytokines related to autophagy, apoptosis, pyroptosis, and inflammatory response; and lead to cytotoxic damage. (5) ROS Inhibitors: NAC, Vitamin E, acetyl L-carnitine, etc

Under physiological conditions, a balance exists between oxidants and antioxidants (e.g., catalase, Superoxide dismutase, Glutathione, and vitamin C/E) in cells. This state plays an important role in maintaining cell growth and regulating cell functions. After entering the cells, AgNPs slowly release Ag^+^ in the cytoplasm or organelles of cells. In particular, lysosomes with low pH are more conducive to the release of silver ions. As the energy center of cells, mitochondria are organelles essential for cell survival. They become the target of AgNPs because of sulfhydryl (–SH) molecules on the cytoplasm and the inner membrane of mitochondria, so Ag^+^ is released by AgNPs; these AgNPs interfere with the permeability of the mitochondrial membrane and disrupt the function of mitochondria, resulting in the continuous production and accumulation of ROS in these organelles, eventually causing protein denaturation, DNA damage, lipid oxidation, and irreversible cell damage [24, 25]. Excessive ROS in cells can also cause ER stress and lysosomal dysfunction, which eventually cause cell damage. In addition, ROS is an important signal molecule that plays an important role in the signal transduction of autophagy, apoptosis, and inflammatory response. In in vivo and in vitro studies, ROS inhibitors or scavengers, such as N-acetylcysteine (NAC) [26], vitamin E [27], acetyl L-carnitine (ALC) [28], supplements with zinc [29], and addition of rutin [30], can effectively reduce intracellular oxidative stress responses and protect cells from cytotoxicity induced by AgNPs. Although studies have demonstrated that the cytotoxicity of AgNPs is mainly mediated by ROS-induced autophagy, apoptosis, and inflammation, the mechanism of cytotoxicity induced by AgNPs is poorly understood (Fig. 2) [23, 31].

### Autophagy

Autophagy is considered one of the main mechanisms for nanomaterials to cause cytotoxic damage. Jin Hou et al. [32] found that ROS in breast epithelial cells increased after AgNPs were treated for 24 h, and ROS production was significantly reduced after treatment of cells with antioxidant NAC, and they observed the formation of autophagosomes and autophagic vacuoles containing part of the cytoplasm to be degraded by transmission electron microscopy (TEM). AgNPs induce cellular autophagy by inducing the production of intracellular ROS and then activate signaling pathways including PI3K-AKT-mTOR, AMPK-mTOR and MAPK (JNK, p38 and ERK). In the process of autophagy, the expression of autophagy-related marker proteins LC3-I and LC3-II increased and the ratio of LC3II/LC3-1 and Beclin-1/β-actin increased significantly, the ratio of p62/β-actin decreased significantly [33, 34]. In addition, AgNPs can activate transcription factors such as NF-κB and Transcription factor EB (TFEB), which can up-regulate the expression of autophagy-related proteins and increase the probability of autophagy [35–37]. Importantly, a study in vitro found that the autophagy inhibitor 3-methyladenine (3-MP) inhibits the autophagy response induced by AgNPs [38].

### Apoptosis

Apoptosis is one of the ways of programmed cell death, mainly including mitochondrial pathway (endogenous apoptosis pathway), death receptor pathway (exogenous apoptosis pathway), and ER stress pathway. After penetrating cells, AgNPs are continuously deposited in mitochondria, causing the continuous generation of ROS, resulting in mitochondrial damage and dysfunction, releasing proapoptotic active protein and cytochrome-c into the cytoplasm, activating Caspase-3/9, and inducing apoptosis [8, 39–41]. In addition, AgNPs can induce apoptosis by activating endogenous apoptosis-related MAPK molecules, including *p38*, *JNK*, and *ERK* [42, 43]. AgNPs can also trigger the production of ROS in cells, cause death receptors (FAS and TNFRs) to bind to ligands, and activate caspase-8; they initiate the downstream caspase cascade pathway to stimulate cell apoptosis [44]. Xue et al. [39] found that AgNPs cause mitochondrial damage by inducing ROS generation in human liver cancer cells (HepG2), downregulating NF-kB, and activating Caspase-3/8, and activating cell apoptosis mediated by the Fas death receptor pathway. ER serves as the main storage of intracellular Ca2^+^, ROS induced by AgNPs causes ER stress, and some Ca^2+^ in the ER become transferred to mitochondria to further trigger the activation of the mitochondrial apoptosis pathway. Ca^2+^ can also activate pro-caspase-12 in the ER membrane to generate caspase-12 and further stimulate caspase-3 to mediate apoptosis [45, 46]. The induction of cell apoptosis by AgNPs is a complex process, and three different pathways may be connected to one another to promote cell apoptosis and produce toxic effects.

### Pyroptosis and inflammatory response

Cell pyroptosis induces the formation of membrane pores with an inner diameter of about 10–14 nm by cleaving Gasdermin (GSDM) through inflammatory body-activated caspase-1 (classical pathway) and bacterial lipopolysaccharide (LPS)-activated caspase-4/5/11 (nonclassical pathway). These membrane pores cause cell swelling, cytoplasmic outflow, and even cell membrane rupture, which eventually triggers cell pyroptosis, accompanied by caspase activation of the proinflammatory factors IL-1β and IL-18 are formed and released through membrane pores to recruit inflammatory cells to mediate high-intensity inflammatory responses [47–49]. AgNPs induce cellular ox lysosomal disorders to further activate the inflammatory body complex composed of NOD-like receptor (NLR) family, pyrin-domain-containing 3 (NLRP-3), apoptosis-associated speck-like (ASC) adapter protein, and Pro-caspase-1; this complex mediates caspase-1 activation, which not only promotes the formation and release of IL-1β and IL-18 to mediate inflammatory responses but also cleaves GSDM to allow the N-terminal domain of GSDMD to form pores in the plasma membrane and eventually lead to cell pyroptosis [50, 51]. AgNPs induce the rapid ER stress response of ER ATF-6 sensor degradation, resulting in the activation of NLRP-3 inflammasome regulated by Caspase-4 in cells [52]. Inflammation is accompanied by the release of common cytotoxic damage and proinflammatory cytokines, including IL-1β, IL-4, IL-6, TNF-α, IFN-γ, chemokines, transforming growth factor-ß (TGF-ß), and prostaglandin E2 (PGE2). These proinflammatory cytokines cooperate with one another to recruit, stimulate, and activate immune cells and thus participate in local inflammatory responses and cause cytotoxic damage [53]. Nerve cells (e.g, microglia, cerebral microvascular endothelial cells (ECs), and neurons) [54, 55] and immune cells (e.g., monocytes, macrophages, and neutrophils) [56–58] exposed to AgNPs increase the secretion of pro-inflammatory cytokines and enhance inflammatory responses. Similarly, by testing the serum of animal models exposed to AgNPs, these proinflammatory cytokines increase significantly, which is closely related to the dose and physical properties of AgNPs [59–61].

## Toxic injury to the BBB

Although only a small part of AgNPs can be transported across the BBB and enter brain tissues because of the limitation of the BBB, these AgNPs experience difficulty in entering the brain to be eliminated because of the lack of an effective elimination mechanism. Even if an organism ingests low-dose AgNPs, they can cause neurotoxicity after long-term accumulation [62]. The intake of AgNPs is more susceptible to oxidative stress reactions, which can accelerate the neurotoxicity of organisms, because of the high fat content of brain tissues, low regeneration capacity, and high energy consumption of brain tissues [63].

### The mechanism of AgNPs transported across the BBB

**Fig. 3:**
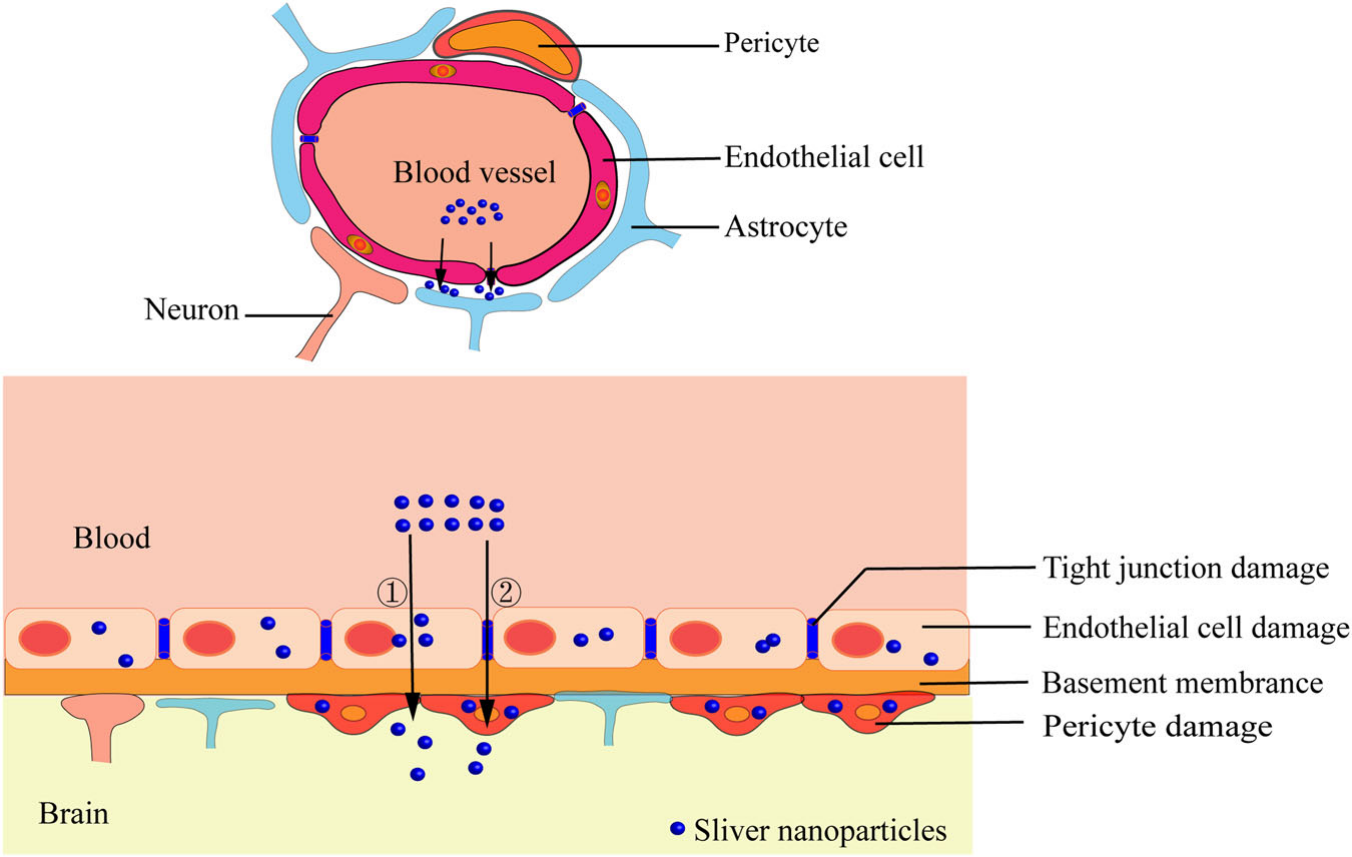
Mechanism of AgNPs transported across BBB. (1) The BBB is composed of ECs, basement membrane, TJs between ECs, astrocytic end feet, and pericytes; (2) Pathway of AgNPs that have entered the blood circulation transported across the BBB: ① Transendothelial cell pathway, ② paracellular transport pathway; (3) The ingestion of AgNPs by ECs, astrocytes and pericytes causes damage of these cells, resulting in the structural damage and dysfunction of BBB, so as to accelerate the process of AgNPs transported across BBB

The BBB, which is mainly composed of ECs, basement membrane, tight junctions (TJs) between ECs, astrocytic end feet, and pericytes, can effectively protect brain tissues from the damage of exogenous substances and pathogenic microorganisms and play an important role in maintaining the stability of the brain microenvironment. Substances are transported across the BBB mainly through two pathways: the transendothelial cell pathway, including passive and active mechanisms, and the paracellular transport pathway regulated by TJs between ECs in the CNS [64]. After binding to serum proteins in blood flow, AgNPs reach the BBB area with blood flow, they can pass through ECs and enter brain tissues via the transcellular pathway, such as passive diffusion, carrier-mediated active transport, endocytosis, and pinocytosis [65]. In addition, some AgNPs with a small particle size may be transported across the BBB through the paracellular pathway, that is, passing through a 4–6 nm gap at the TJs formed between ECs [64]. Interestingly, AgNPs can be transported directly to the CNS through the olfactory or trigeminal nerves [66]. Some scholars observed the BBB model in vitro treated with AgNPs via TEM and found that most AgNPs accumulate in the cytoplasmic vacuoles of ECs; furthermore, the number of AgNPs containing vacuoles in astrocytes is significantly less than that in ECs possibly because only a small number of AgNPs can continuously migrate to astrocytes through ECs (Fig. 3) [67].

### Toxic injury of AgNPs to the BBB

When AgNPs are ingested by ECs, pericytes, and astrocytes in the transport across the BBB, they can cause an oxidative stress reaction in these cells, cause dysfunction, death of these cells, and eventually lead to the increased permeability or integrity of the BBB, which is more conducive to the entry of AgNPs into the CNS to induce neurotoxicity [68].

#### Damage to ECs and intercellular TJs

ECs in the BBB are tightly adhered with each other through TJs, which are composed of transmembrane proteins (mainly including Claudins-1/3/5/12 and Occludins), junctional adhesive molecules (JAM-1/2/3), cytoplasmic attachment proteins (Zonula Occludens (ZO)-1/2/3), cytoskeleton proteins (Fibros-actin) and other proteins, forming a continuous physical barrier to effectively prevent the paracellular diffusion of substances in the blood into the CNS [69, 70]. Compared with peripheral vascular ECs, ECs in the BBB are less susceptible to AgNPs-induced toxic injuries. This may be due to more mitochondria and fewer plasma membrane vesicles within ECs in the BBB or the presence of special cellular components that can attenuate toxic injuries caused by AgNPs [64, 71]. AgNPs-induced toxic injuries of ECs and the opening or closing of TJs caused by downregulation of TJs-related proteins between these cells, which may ultimately alter the structural integrity and permeability of the BBB. An in vitro BBB mode constructed by co-culture of human brain microvascular ECs and human astrocytes exposed to AgNPs (PVP coating, 50 nm, and 10 μg/ml) has shown no significant change in ECs viability after AgNP treatment for 24 h; after 48 h, cell viability decreases significantly by 20%. Most of these AgNPs accumulate in ECs, while some of them are in astrocytes. After 24 h of exposure, C9 and other inflammatory pathways in ECs are significantly activated, whereas the expression of proteins involved in BBB injury and anti-inflammatory responses increases after 48 h of exposure. These results show that exposure to AgNPs can activate the inflammatory response and oxidative stress of ECs in early stages and induce the expression of protective proteins in later stages to weaken the toxic effects of AgNPs [67]. In addition to direct injury to ECs, AgNPs can also cause BBB toxic injury by downregulating the expression of TJs-related proteins between ECs. Liming et al. [72] established a BBB model by triple coculture of rat brain microvascular ECs, pericytes, and astrocytes. They demonstrated that TJs between ECs is discontinuous, and the expression of the TJs-related protein ZO-1 decreases after 24 h of exposure to AgNPs, resulting in a significant increase in the BBB permeability. TEM has also revealed severe atrophy, vacuolization, and ER dilation in astrocytes, and the lesions may be caused by AgNPs inhibiting the antioxidant defense of astrocytes and inducing astrocyte inflammation and apoptosis. In vivo studies also found that after intravenous injection of AgNPs (7 nm, 5 mg/kg, CT coating) into 16 weeks old rats for 24 h, the expression of TJs-related protein Claudin-4 was significantly reduced, and the permeability of the BBB didn’t change significantly due to the short experimental time [73].

#### Disruption of the defensive and restorative effects from astrocytes/pericytes in BBB injury

Astrocytes participate in the formation of the BBB through end-feet extended from astrocytes which wrapped around the surface of ECs. These cells exchange substances and signals with the ECs to regulate the structural integrity and permeability of the BBB, which play vital role in protecting and repairing the BBB in the process of BBB injury caused by ischemia and hypoxia [64]. After inducing astrocytes ablation in the brain of experimental animals by drugs (3-chloropropanediol, tamoxifen, etc.), the expression of ECs-related TJs protein ZO-1 in brain decreased significantly, and accompanied by a significant increase in the permeability of BBB. The injury of astrocytes ablation to the BBB can be repaired by re-introducing astrocytes which induce the TJs related protein re-expressed and the integrity of the BBB re-stored during the repair process. It is suggested that pericytes are not sufficient to compensate for BBB injury caused by astrocytes ablation during the repair process of astrocytes [74, 75]. Pericytes wrap around the surface of ECs during the formation of the BBB, studies have confirmed that these cells play vital role in the process of integrating regulatory information from ECs and astrocytes to establishing the complete structure and function of the BBB, and the crosstalk between pericytes and ECs considered more important than that between astrocytes and ECs [76, 77]. Bhowmick et al. [78] discovered that the expression levels of peripheral cell markers, including platelet-derived growth factor receptor β (PDGFRβ), NG2, and CD13, were considerably decreased following traumatic brain injury (TBI) induced by in vivo percussion injury in mice. Additionally, the expression of TJ-related proteins such as occludins, Claudin-5, ZO-1, and JAM-1 were also downregulated, leading to a significant increase in BBB dysfunction, which greatly enhanced the permeability of sodium fluorescein and tracer Evans blue. The impairment of pericyte-endothelium crosstalk caused by the disruption of the PDGFR-β/PDGF-β signaling pathway in TBI is responsible for the loss of interaction between pericytes and ECs, thereby leading to BBB dysfunction and is considered one of the pathogenic mechanisms of TBI. Under conditions of severe and long-term oxygen deprivation, the role of pericytes in protecting and repairing the damaged BBB is more significant compared to the role of astrocytes [79].

During the process of AgNPs transported across the BBB, the crosstalk between astrocytes/pericytes and ECs plays a crucial effect in protecting and repairing the BBB injury caused by AgNPs. In instances where this effect is inadequate and fails to meet the requirements for the repair of BBB injury caused by AgNPs, BBB dysfunction may ensue. Nevertheless, the precise mechanism behind this process remains elusive. Dabrowska et al. [80] fed adult rats with a solution of AgNPs (particle size of 10 nm, citric acid coating, 0.2 mg/kg) via oral administration for 2 weeks and found that astrocytes and neurons around blood vessels in the cerebral cortex and hippocampus are edematous. Although pericytes have no significant changes in ultrastructure, the expression of the pericyte marker PDGFRβ has changed. RT-PCR results have indicated that the mRNA levels of claudin-5, ZO-1, and Occludin among ECs decrease. Under the same experimental conditions, 2-week-old rats exposed to AgNPs for 14 days exhibit pathological changes mainly characterized by enhanced cerebral microvascular permeability and local edema of perivascular astrocytes and peripheral nerve fibers. Electron microscopy observation of the endocytotic activity of ECs with few pinocytic vesicles and without depression has not shown obvious abnormal changes in pericytes and TJs complex between ECs. Interestingly, the relative levels of connexin proteins ZO-1 and Claudin-5 are reduced by 40% and 30% compared with those of rats without exposure to AgNPs; the Occludin level is significantly reduced by about 20%; the mRNA expression levels of ZO-1 and Claudin-5 are five times higher than those of rats that are not exposed to AgNPs; the expression level of the pericyte marker PDGFβR protein does not change significantly compared with rats without exposure to AgNPs; however, the mRNA expression level of the PDGFβR protein increases significantly, especially the mRNA overexpression of the astrocyte-specific marker GFAP and the ECs adhesion molecule ICAM-1, which may explain the toxic mechanism of BBB injury induced by AgNPs [81]. In addition, Dan et al. [73] intravenously injected a solution containing AgNPs (7 nm, citric acid coating, 5 mg/kg) into 16-week-old rats. After 24 h, they found that Ag^+^ is present in the brain tissue of rats exposed to AgNPs but not in the brain tissue of rats exposed to Ag^+^. Interestingly, AgNPs did not cause significant changes in the permeability of BBB. However, the expression of Claudin 4 decreases significantly. These results imply that short-term exposure to low-dose AgNPs does not cause BBB dysfunction. However, prolonged exposure to low-dose AgNPs will inevitably cause structural disruption and dysfunction of the BBB, especially for young organisms with more severe injury. As such, AgNPs can easily enter the brain and accumulate to induce neurotoxic reactions. Therefore, AgNPs should be cautiously added to dental restoration materials for clinical restoration treatment.

## Neurotoxicity mediated by AgNPs

**Fig. 4:**
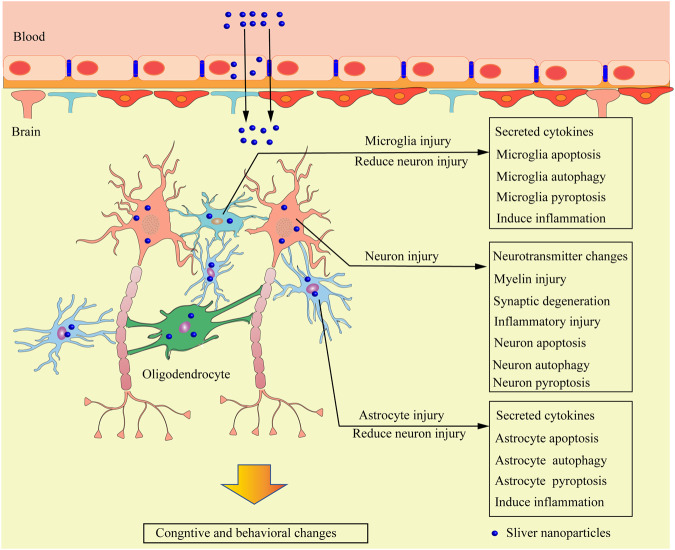
Mechanism of neurotoxicity caused by AgNPs. The uptake of AgNPs by neurons, astrocytes, and microglia causes damage to these cells. Non-neurotoxic levels of AgNPs uptake by astrocytes and microglia can protect neurons from toxic injury, but AgNPs can damage and cause dysfunction of astrocytes and microglia under the neurotoxic level of AgNPs, leading to the loss of the neuronal protection of glial cells. AgNPs can also cause neuronal injury and degeneration. Consequently, these changes alter the cognition and behavior of organisms

Nerve cells in the brain are divided into two categories: neurons and glial cells. The formation of complex synaptic connections between a large number of neurons is an important basic process in the formation and maintenance of brain functions, such as emotions, learning, memory, and sports. Glial cells participate in the formation of the myelin sheath of neuronal axons, support neuronal metabolism and signal transduction in the brain, and protect neurons from toxic injury. After penetrating the BBB, AgNPs can enter neurons and glial cells, mediate the oxidative stress reaction of cells, cause the disorder of the structure and function of these two types of cells, even induce the programmed cell death of cells, and finally cause neurotoxicity (Fig. 4).

### The effect of AgNPs on nerve cells

#### Neurons

Neurotoxic substances induce toxic effects by inducing changes in neuronal structure or viability [82], mainly including affecting neuronal viability, neuronal axon myelin sheath, or synaptic transmission process. Neuronal necrosis or apoptosis induced by toxic substances is usually the final result of the toxic injury. In studies on the cytotoxic effect of AgNPs on neurons, a specific brain region or neuronal cell line that synthesizes and secretes a specific neurotransmitter is mostly selected. Ma et al. [40] exposed hippocampal neurons cultured in vitro to AgNPs and found that the mitochondrial membrane of neuronal cells is polarized, and the concentration of ROS in the mitochondria increases; the caspase-3 dependent apoptosis pathway is eventually activated to induce hippocampal neuronal apoptosis. They also found that the membrane potential of mitochondrial depolarization stabilizes after selenium treatment of hippocampal neurons exposed to AgNPs, thereby preventing the accumulation of ROS and the activation of caspase-3. These results show that selenium can protect neurons and reduce the neurotoxicity induced by AgNPs. Kursungoz et al. [83] sliced the rat hippocampus and exposed it to AgNPs for 1 h and observed that the survival rate of hippocampal cells is significantly reduced in a dose-dependent manner. They examined the specimens through TEM and demonstrated that AgNPs are distributed in the extracellular matrix, and only the larger AgNPs enter neurons via the phagocytosis pathway. In an in vitro study, AgNPs (1–50 μg/ml) can cause neuronal mitochondrial dysfunction and loss of cytoskeleton proteins (β-tubulin and filamentous actin [F-actin]) to inhibit the growth of neurons and reduce the vitality of neurons [84]. Apart from the above changes in neurons exposed to AgNPs, the ultrastructure of neuronal synapses changes after long-term exposure to AgNPs, and the levels of Synapsin-I and Synaptophysin, which are presynaptic proteins, and postsynaptic receptor density protein95 (PSD-95), which is an indicator of postsynaptic densities, also significantly decrease; these results indicate that AgNPs can cause the abnormal structure and function of neuronal synapses [84, 85]. The ingested AgNPs in neuronal cells and the released Ag^+^ continuously accumulate in organelles, such as lysosomes and axoplasmic mitochondria, causing cell dysfunction, inhibiting the release of neurotransmitters, down-regulating neurotransmitter levels in the brain, and damaging the myelin sheath of neuronal axons [86, 87]. AgNPs can also alter the action potential of neurons by inhibiting the voltage-gated sodium currents of neurons and affect the information transmission process of neurons [88]. Therefore, after being ingested into neurons, AgNPs affect the structures of neuronal synapses and myelin sheath, neuronal neurotransmitter release, and cell action potential. As a result, neuronal dysfunction occurs, and even neuronal apoptosis or necrosis is directly induced. Thus, neurotoxic reactions, such as biological behavior and cognitive impairment, occur.

#### Glial cells

Glial cells in the CNS are mainly divided into three types: astrocytes, oligodendrocytes, and microglia. Studies have shown that the ingestion of AgNPs in the brain does not directly damage neurons, but AgNPs can induce cytokines (e.g., interleukin, TNF-α, and MCP-1) produced and secreted by glial cells, which can cause toxic injury to neurons [89]. These observations occur probably because glial cells in the brain of organisms exposed to AgNPs are more sensitive than neurons even in the presence of low doses of AgNPs [90].

Astrocytes are glial cells with the largest number and the most complex functions in the brain; they can regulate neuronal growth and guide neuronal migration and synapse formation, BBB formation, substance metabolism, extracellular K^+^ concentration stabilization, neuronal neurotransmitter regulation, and CNS homeostasis maintenance [91, 92]. Astrocytes and neurons regulate the activity and metabolism of neurons through close contact. After being exposed to AgNPs, cells play a detoxification effect by activating the antioxidant system and chelating Ag^+^ released by AgNPs. When the detoxification effect of astrocytes is insufficient or ineffective, neuronal death or dysfunction often occurs mainly depending on the exposure time and dose of AgNPs [65]. Luther et al. [93] incubated the in vitro cultured rat primary astrocytes with AgNPs (10 μg/ml PVP coating) for 24 h and found that the accumulation of AgNPs in astrocytes is time and concentration dependent. Interestingly, the accumulation of AgNPs does not damage cell viability or reduce glutathione contents in cells, indicating that PVP coating can effectively reduce the toxic effect induced by AgNPs. They also demonstrated that incubation at 4 °C can reduce the accumulation of AgNPs in cells by 80% compared with that at the experimental incubation temperature of 37 °C. These results suggest that a low temperature may be one of the factors that reduce the accumulation of AgNPs in astrocytes to reduce toxic injury. Astrocytes are often affected by different AgNPs concentrations with various outcomes. For instance, cytotoxic AgNPs induce changes in ROS in astrocytes depending on time and concentration; thus, programmed cell death occurs. However, exposure to AgNPs at a noncytotoxic level often increases the secretion of a variety of cytokines in astrocytes and participates in neuroinflammation [42]. Studies have shown that astrocytes can be detoxified by AgNPs that up-regulate the expression of metallothionein in astrocytes. Metallothionein contains a large number of –SH combined with Ag^+^ released by intracellular AgNPs and stores them in astrocytes to prevent it from being released from astrocytes again. This protein can also combine with ROS produced under oxidative stress induced by AgNPs to enhance the antioxidant defense mechanism of cells and elicit a synergistic protective effect [94, 95].

As immune cells in the CNS, microglia remove foreign bodies and pathogenic microorganisms that invade the CNS. Inflammatory response after overactivation are closely related to neurodegenerative diseases [96]. Gonzalez et al. [97] incubated mouse microglia N9 cell lines with AgNPs and found that they absorb AgNPs, which in turn are internalized and dissolved to release Ag^+^; consequently, microglia become activated to express hydrogen sulfide (H_2_S) enzyme and chelated Ag^+^ released by AgNPs to form nonreactive silver sulfide (Ag_2_S) to reduce the toxicity of AgNPs. In this study, they also treated microglia with lipopolysaccharide (LPS, 500 ng/ml) and AgNPs for 1 h and then continued culturing for 24 h. They showed that the contents of the pro-inflammatory microglia markers ROS, NO, and TNF-α decrease, further indicating that H_2_S produced by microglia can be used as an effective anti-inflammatory agent to attenuate the inflammatory response mediated by microglia, reduce the inflammatory injury of adjacent neurons, and achieve a certain neuroprotective effect. Sikorska et al. [98] observed that the viability and phagocytosis of mouse BV-2 microglial cells treated with AgNPs (50 μg/ml) were inhibited, and the clearance of β-amyloid protein were attenuated, which is more conducive to the occurrence and rapid development of Alzheimer’s disease (AD). Therefore, the intake of AgNPs by patients with AD may accelerate disease development, which needs further studies.

### Main factors affecting neurotoxicity of AgNPs

AgNPs can transfer from the blood to the brain and accumulate in the brain over time regardless of their contact pathways. The particle size, shape, surface coating, contact time, dose, intake pattern, Ag^+^ release rate, and interaction with proteins are the key factors determining the neurotoxicity of AgNPs and their distribution, accumulation, and elimination in the brain [65, 99, 100]. In vivo and in vitro studies have shown that AgNPs-induced neurotoxicity is dose and time dependent. Nakkala et al. [101] orally fed rats with AgNPs (5 and 10 mg/kg/day) for 29 days. They found 3.043 μg/g tissue Ag^+^ that accumulated in the brain of the rats treated with 10 mg/kg/day but detected no accumulation of Ag^+^ in the brain tissue of the rats treated with 5 mg/kg/day. Interestingly, they observed the complete removal of Ag^+^ in the brain tissue the 89th day after the intake of AgNPs was terminated. They also did not find the presence of Ag^+^ in the brain tissue exposed to low-dose AgNPs, possibly because of the insufficient sensitivity of the detection method and the insufficient exposure time. Lin Li et al. [102] also demonstrated that AgNPs accumulated in the brain tissue of rats fed with different doses of AgNPs (32, 80, or 200 mg/kg/day) for 28 days. They also observed that a large part of neurons in the prefrontal cortex has obvious apoptotic pathological changes, such as loss of cell integrity, cytoplasmic atrophy, and blurred nuclei. These pathological changes worsen as the dose of AgNPs increases. Although AgNPs induce oxidative stress and cause toxic injury, they initiate a self-protection program by upregulating the transcription of antioxidant genes in cells. Rahman et al. [103] detected the caudate nucleus, frontal cortex, and hippocampus after the intraperitoneal injection of AgNPs at different doses (0, 100, 500, and, 1000 mg/kg) into adult mice for 24 h. Oxidative stress and antioxidant-related genes, such as *Fmo2, Gsr, and Txnip*, are significantly and differentially expressed. *Fmo2* is significantly upregulated in the frontal cortex, caudate, and hippocampus. Its expression in the caudate nucleus is upregulated by 94.79 times at a dose of 500 mg/kg. Conversely, its expression levels in the frontal cortex and hippocampus are upregulated by only 2.16 and 1.84 times at the same dose, respectively. At the dose of 1000 mg/kg, its expression is increased by 4 times. Therefore, the degree of oxidative stress caused by AgNPs in various brain regions is different. Long-term exposure to high-dose AgNPs often causes serious neurotoxic reactions in a short time. Notably, the developmental status of organisms is also an important factor affecting the neurotoxicity of AgNPs. Related studies have reported that the gastrointestinal tract of juvenile animals exposed to the same dose of AgNPs absorbs AgNPs to a greater degree than that of adult animals. The silver content in the serum of juvenile organisms is significantly higher than that of adult organisms; furthermore, the amount of silver deposited in the brain of juvenile organisms is higher than that of adult organisms, indicating that more severe neurotoxicity occurs in the brain of juvenile animals [81]. Sharma et al. [104] compared the neurotoxicity of AgNPs to mice of different weeks and found that the smallest and oldest mice showed greater neurotoxicity than the middle-age group. Antsiferova et al. [105] also conducted a study to compare the variations in behavioral tests, such as locomotor activity, exploration behavior, and anxiety assessment, in mice treated with AgNPs for 60 days at the age of 2 and 5 months. It was observed that elder mice adapted to the neurotoxicity of AgNPs better than the younger mice, while younger mice exhibited more drastic anxiety than the elder ones. As shown above, the neurotoxicity decrease of AgNPs with aging manifested in the improvement of certain behavioral functions of elder organisms in comparison with younger ones, the increase of adaptive homeostasis quality with age in the period of vitality blossom, and suggested that infants, children, and the elderly easily suffer from neurotoxic injury when ingesting AgNPs. More importantly, AgNPs can even cross the placental barrier to affect embryonic development. In pregnant rats exposed to AgNPs (20 and 50 nm), the silver content in the hippocampus of offspring male rats increases. Pathological changes such as shrinking of hippocampal somatic cells, increase in the intercellular space, and decrease in the number of Nissl bodies, occur. In the Morris water maze experiment, their escape latency is also extended. These findings further indicate that AgNPs can reduce the spatial cognitive ability of rat offspring and cause cognitive impairment [106]. Therefore, dental restoration materials containing AgNPs should be cautiously given to special populations such as pregnant women, infants, children, and the elderly.

### Changes of toxic injuries induced by AgNPs

#### Mechanism of biological behavior changes induced by AgNPs

##### Changes of neurotransmitters

Neurons in the CNS carry out information transmission and communication through a variety of neurotransmitters, mainly including acetylcholine, biogenic amines (e.g., dopamine, norepinephrine (NE), epinephrine, and 5-hydroxytryptamine (5-HT)), amino acids (e.g., glutamate and γ-aminobutyric acid), and peptides (e.g., neuropeptides). These neurotransmitters are essential for the integration of multiple functions of the CNS, including learning, memory, exercise, mood, sleep, and hormone regulation [107]. AgNPs cause neuronal dysfunction or apoptosis through oxidative stress, thereby altering neurotransmitter levels. In an in vivo study, Hadrup et al. [44] found that the concentration of dopamine in the brain of rats decreases after the oral administration of AgNPs (14 nm, 4.5 and 9 mg/kg/day) for 14 days, but it increases after 28 days. At 9 mg/kg/day, the concentration of 5-HT in the brain of rats increases after oral administration for 28 days. Similarly, Skalska et al. [108] found that the concentration of dopamine in the brain of rats decreases after 14 days of oral administration of AgNPs (2.25 and 4.5 mg/kg body weight, PVP coating) but increases after 28 days. The concentration of 5-HT in the brain of rats increases at a dose of 9 mg/kg AgNPs. Conversely, Attia et al. [87] found that the concentrations of acetylcholinesterase, dopamine, and serotonin in the brain of mice are significantly insufficient after the oral administration of 100 and 1000 mg/body weight AgNPs (26.9 nm, CT coating) for 28 days. These results suggest that the level of neurotransmitters is affected by many factors, such as the physical characteristics of AgNPs, exposure time, and dose. Dopamine concentration decreases in the early stage of biological intake of AgNPs, possibly because of the apoptosis of dopaminergic neurons in the brain caused by early AgNPs. By comparison, the increase in the later stage may be caused by the protective increase in the dopamine neurotransmitter production of surviving dopaminergic neurons. 5-HT changes when the dose is high and the exposure time is too long, likely because serotonin neurons are more tolerant to the toxic effect of AgNPs.

##### Myelin injury

Myelin sheaths formed by myelin are wrapped around neuronal axons and implicated in protecting neuronal axons and maintaining neuronal signal transmission. In vivo studies have shown that the oral administration of AgNPs in rats significantly changed the oxidative stress, neurotransmitters, and amino acids in the brain, consequently causing pathological changes such as astrocyte proliferation and neuronal demyelination [30, 87]. The long-term exposure of adult rats to low-dose CT-stabilized AgNPs (10 nm, 0.2 mg/kg/weight) causes hyperalgesia, and the expression levels of myelin-specific proteins, namely, CNP, MAG, and MOG decrease, resulting in the disorder of myelin formation in the brain of rats [109]. Dąbrowska et al. [110] exposed adult rats to AgNPs (10 nm, 0.2 mg/kg) for 2 weeks and found that the concentration of protein and nonprotein sulfhydryl groups in the myelin structure of the brain tissue decreases, whereas lipid peroxidation increase. The expression of superoxide dismutase (a free radical scavenger) and the inefficient processing of protein glutathione (Cell protection mechanism to prevent irreversible oxidation) increase. The oxidative stress mediated by AgNPs results in the disorder of the ultrastructure of the myelin sheath in the brain, thereby damaging the normal structure of the myelin sheath or inhibiting the formation of myelin sheath. Neurons lacking myelin sheath protection often cannot effectively transmit information and eventually cause abnormal brain function.

##### Synaptic degeneration

The synaptic structure of neurons is an important structure involved in information transmission and signal transduction in the CNS. An in vitro study has shown that AgNPs (1–50 μg/ml) significantly reduce the expression of neuronal presynaptic proteins (Synapsin-I and Synaptophysin) and PSD-95 and negatively affect neuronal development, physiological functions, and signal synaptic transmission [84]. In some similar studies, Skalska et al. [85] exposed rats to AgNPs (10 nm, citric acid coating) for 2 weeks and observed that the synaptic structure becomes blurred, and the synaptic vesicles in the center of the presynaptic zone are dense. The most characteristic change is the disorder of the synaptic membrane that leads to the release of synaptic vesicles into the nerve fiber membrane. The levels of presynaptic proteins (synaptophysinI and synaptophysin) and PSD-95 decreased significantly. These changes mainly occur in the hippocampal region of the brain, further suggesting that the synapses of neurons in the hippocampal region are more sensitive to AgNPs. Repar et al. [90] exposed neurons and astrocyte cocultures to AgNPs (20 nm, CT coating) and found that the expression of PSD-95 and synaptophysin is downregulated in neurons, leading to neuronal degeneration. The degrees of downregulation and degeneration depend on the concentration of AgNPs. Therefore, AgNPs cause the synaptic degeneration of neurons, which are characterized by a decrease in protein expression levels related to synaptic ultrastructure and synaptic structure. Neurotransmitter transmission disorder even causes neuronal degeneration.

#### The influence of AgNPs on biological behavior and cognition

AgNPs pass through the BBB and accumulate in neurons and glial cells in the brain, inducing oxidative stress and causing neuronal and glial cell dysfunction, programmed cell death, and biological behavior and cognitive changes [10]. Greish et al. [111] injected 0–3 times of AgNPs intravenously (0.1 ml each time, once a week) in adult mice (8–10 weeks old) and found that the social interaction and exploratory activities of the mice decrease, and their memory, learning, and motor functions are impaired. Studies have also shown that the effects of AgNPs on mammalian behavior and cognitive function has three different stages: anxiety, activation of the adaptive mechanism, and the influence of cognitive functions [112]. To study the causes of the effects of AgNPs on mammalian behavior and cognitive function, Antsiferova et al. [113] conducted neutron activation analysis and histological analysis to examine the accumulation of silver in the hippocampus, cerebellum, cortex, and other brain tissues of male mice after the oral administration of PVP coating AgNPs every day for 30, 60, 120, and 180 days. They found a step-like increase in the silver content in the hippocampus, cerebellum, cortex, and other brain regions. The step-like increase in the hippocampus, cerebellum, and cortex is visible on the 120th day, and the similar phenomenon in other brain tissues is visible on the 180th day. Nissl staining revealed that the density of neurons in the hippocampal CA2 region of mice exposed to AgNPs for 120 and 180 days decreases and becomes arranged in a scattered manner. However, no similar changes occur in other brain regions in all the tested stages, indicating that the long-term memory impairment of mice may be attributed to the accumulation of silver in the whole brain and its subregions, thereby continuously causing neuronal injury in the CA2 region. Notably, the accumulation and distribution of silver in various regions of the brain are uneven; in other brain regions, such as the cortex and cerebellum, no pathological changes similar to those in the hippocampal CA2 region are observed, suggesting that the hippocampal region in our brain is more sensitive to the toxic effect of AgNPs. Studies have also shown that the loss of the hippocampal CA2 region can cause damage and impairment of long-term memory function [114]. The stem cells and progenitor cells in the hippocampus of adult mammals divide to form new neurons, which may be involved in learning and memory, anxiety and stress regulation, and social behaviors of organisms [115]. In vitro studies have shown that the increased active oxygen of neural stem cells exposed to AgNPs inhibits the proliferation and differentiation of neural stem cells, induces a decline in the viability of stem cells, and ultimately leads to apoptosis [28, 116]. Therefore, AgNPs may affect the behavior and cognitive function of the hippocampus by interfering with the formation of neurons in this brain region. The formation of neurons in the hippocampus is involved in behavior and cognition and accompanied by the enhancement of neuronal synaptic plasticity [117]. AgNPs can change the spatial cognition and spatial reference memory of rats by affecting the synaptic plasticity of the hippocampus [118, 119].

The cerebellum is the motor regulatory center that maintains body balance and coordinates random movement. In vivo studies have shown that AgNPs can damage cerebellar cortical cells by inducing oxidative stress, inflammatory response, and apoptosis. They can also cause the disorder of intracellular Ca^2+^ by downregulating the expression of calmodulin [59]. After suffering from cerebellar damage caused by AgNPs, organisms often have motor function damage, such as ataxia and coordination disorders. Yin et al. [120] exposed newborn SD rats to AgNPs with a particle size of 20 nm for 14 weeks via intranasal infusion and observed that the rats developed cerebellar ataxia (impairment of motor performance and coordination function). In this study, AgNPs destroy the cerebellar cortical granular layer, activate glial cells, and reduce the expression level of cerebellar calcium channel proteins, but they do not alter the expression of potassium channel proteins. Conversely, Dąbrowska et al. [109] exposed adult rats to AgNPs (0.2 mg/kg/day, CT coating) through gastric tube administration for 14 days. Their behavioral tests reveal that AgNPs have no significant impact on the locomotor activity and coordination of adult rats. More importantly, this research group exposed the rats to AgNPs (0.2 mg/kg/day, CT coating) by gastric tube administration from 14 days after birth continuously for 21 days. They evaluated the muscle relaxant or ataxic effects of AgNPs on rats via a Rotarod performance test on the 24th and 52nd days after birth. They found that AgNPs do not influence the locomotor activity and coordination of rat pups [81]. These findings further suggest that the exposure of organisms to low-dose AgNPs is too short to cause damage to cerebellar injury, thereby impairing the coordination and motor ability of organisms.

The continuous accumulation of AgNPs in the brain induces neurotoxic injury to organisms, which is a long-term cumulative effect that occurs even when ingested in low doses of AgNPs. However, the neurotoxic damage caused by AgNPs is not entirely irreversible or preventable. Numerous investigations have revealed that the consumption of AgNPs in isolation by rats elicits toxic injury to the CNS, including cognitive and biological behavior impairments. Notably, co-exposure of rats to AgNPs and either zinc nanoparticles, selenium-loaded chitosan nanoparticles, sulfuric acid, platelet-rich plasma (PRP) or Yttrium Oxide nanoparticles (YO-NPs) can significantly alleviate AgNPs -mediated neurotoxic damage, including the considerable downregulation of oxidative stress reactions in the brain, the Significant improvement of behavioral and cognitive impairments, as well as pathological damage to brain tissue induced by AgNPs [121–125]. All evidence suggests that the addition of these substances in co-addition with AgNPs exhibits a protective effect on neurotoxic damage caused by AgNPs Table 2.

**Table 2:** Summary of neurotoxic injuries of AgNPs in CNS

Types of AgNPs	Animal models	Route, dosage and exposure time of administration	Results	Ref.
PVP-coated AgNPs	Male rats: 140–150 g	*Oral*: 30 mg/kg b.w./day, lasted for 8 weeks	-Significantly changed the level of neurotransmitters and amino acids in brain. ­ Oxidative stress occurs in brain. - The transcriptional levels of NMDA receptors, MAO-A/B and MT-III significant increased. - Astrogliosis and demyelination of neurons accompanied by neuronal degeneration and vacuolation have been observed. - Oral administration of rutin overcomes neurotoxic effects through improving the antioxidant status in brain, correcting the imbalance of neurotransmitters.	[30]
AgNPs: 45–120 nm	Adult male rats: 7–9 weeks old	*ig*.: 10 and 30 mg/kg b.w./day, lasted for 28 days	- The enhancement of Txnip and FMO2 gene expression changed the redox homeostasis in rat brain, Conduciving to activate the inflammatory responses. - Ddit4 and Txnip mRNA levels significantly increased at all exposed dose levels, FMO2 mRNA level highly significant increases in the high-dose group, Ddit4 and JNK protein expression were up-regulated to enhanced apoptosis in the cerebellar cortex. - Presence of considerable amounts of sliver in the cerebellar cortex increased in a dose-dependent manner. -The architecture of the cerebellar cortex were more severely disrupted in the high-dose group than the low-dose group.	[59]
AgNPs: 3–30 nm	Female SD rats: 6 weeks old	*ig*.: 1 and 10 mg/kg b.w./day, lasted for 2 weeks	- Silver content significantly increased to 7.3- and 7.7-fold in the blood and 60- and 312-fold in the brain in the low- and high-dose groups compared to the control group. - Neuronal degeneration, astrocyte swelling, and IL-4 significantly increased in brain even in a low-dose group.	[60]
CT-coated AgNPs: 7 nm	Female rats: 16 weeks old, 280 - 310 g	*i.v*.: 5 mg/kg b.w. AgNPs, 0.0003 mg/kg b.w. Ag^+^ 5% sucrose solution in control group, Rats were sacrificed 24 h after injection	- The permeability of BBB of Rats exposed to AgNPs were not changed, but the expression of Claudin-4 significantly decreased. - Observed astrocyte foot swelling, neuron shrinkage, and AgNPs-like particles in the hippocampus of AgNPs-exposed group. - Ag^+^ was only detected in the hippocampus of rats exposed to AgNPs, while not detected in the control group and the Ag^+^ group, meanwhile, Ag was not detected in the blood after 24 h injection in all groups. - Ca^2+^ signaling pathway and neuroactive ligand-receptors (Grin2a, Drd2, and Adra1d) affected by AgNPs damaged the cognitive and neural development of the brain.	[73]
CT-coated AgNPs: 26.9 nm	Adult male mice: 3 months old, 28–32 g	Oral: 100 and 1000 mg/kg b.w./day, 28 days	- Total Ag^+^ contents in whole brain tissue significantly increased in a dose-dependent manner. - The levels of monoamines (Dopamine and 5-HT) in the brain and the value of enzyme activity of AChE in the cerebral cortex of mice exposed to AgNPs significantly decreased in a dose-dependent manner. - The cerebral cortex of mice exposed to AgNPs showed that neuron pyknosis and increase of neuroglia cells.	[87]
CT-coated AgNPs: 25 nm	Adult male mice	*i.p*.: 0, 100, 500 and 1,000 mg/kg b.w., Mice were sacrificed 24 h after injection	- Changed the expression of genes related to oxidative stress in the caudate nucleus, frontal cortex and hippocampus of mice.	[103]
PVP-coated AgNPs: 8.7 ± 0.4 nm	mice: 2 months and 5 months old	Oral: 50 µg/day, per animal lasted for 60 days	- The behavior of younger and elder mice both exposed to AgNPs showed a decrease in locomotor activity with age. - Elder mice exposed to AgNPs exhibited certain improvements in behavioral functions compared to those of the exposed younger ones. - The silver accumulation levels in the brains of younger and elder mice are equal. - The adaptive homeostasis in CNS exposed to AgNPs non-linearly changes with age increase.	[105]
AgNPs: 10 ± 4 nm,	Adult male rats: 140–160 g	*ig*.: 0.2 mg/kg b.w. /day, once a day, lasted for 14 days	- The level of sliver in serum in the range of 11–13 µg/L, while it was below the detection limit of the applied method in brain homogenates of rats (0.241 mg/kg in solid tissue) after 2 weeks. - The locomotor activity, motor coordination, and memory performance of rats exposed to AgNPs lasting for 14 days didn’t have significant influence. - Brain of rats exposed to AgNPs showed morphological disturbances in myelin sheaths and changed the expression of myelin-specific proteins (CNP, MAG, and MOG), those proteins significantly decreased, while the mRNA level increased.	[109]
PVP-coated AgNPs: 34 ± 2 nm	Male mice: 8 weeks old, 19–27 g	Oral: Suspended AgNPs in distilled water (50 µg/day); lasted for 30 days, 60 days, 120 days and 180 days,	- Mice experienced two adaptation periods to toxic AgNPs, that is anxiety and the development of research instincts, but ultimately failed because AgNPs eventually led to the degradation of long-term memory. - The relatively short (30–60 days) and long administration time (more than 120 days) after exposure to AgNPs is the most dangerous period of brain damage.	[112]
PVP-coated AgNPs: 34 ± 5 nm	Male mice: 8 weeks old	Oral: Suspended AgNPs in distilled water (50 µg/day); lasted for 30 days, 60 days, 120 days and 180 days	- The concentration of sliver in the hippocampus, cerebellum, and cortex at 120 days, and the remaining brain tissue at 180 days increases in a step-like manner. - The irregular and rarefied appearance stratum in the pyramidale and the dispersed arrangement of neurons in the CA2 region in brain were observed. - The long-term contextual fear memory tested 24 h after training of mice in the group after AgNPs-exposed for 180 days were impaired. - Memory and behavioral changes are caused by the accumulation of silver in the brain and neuronal damage to the CA2 subregion of the hippocampus.	[113]
CT-coated AgNPs: 10 nm	Adult male rats: 6 weeks old, 180–210 g	*ig*.: 0.2 mg/kg b.w./day, once a day for 14 days	- The level of ROS, MDA and GPx activity in brain tissue of the group exposed to AgNPs were significantly higher than control group receiving saline. - AgNPs and Ag^+^ were both causing a significant decrease in the reduced-to-oxidized glutathione ratio in brain. -Rats exposed to a very low dose of AgNPs produce mild oxidative stress in their brain but not in the liver, indicating the oxidative stress induced by AgNPs in brain may cause neurotoxicity.	[130]
PVP-coated AgNS, PVP-coated AgNC	Male rats: 7 weeks old	Oral: 3.6 mg/kg b.w./day, lasted for 14 days	Anxiety-like and possibly stereotypical behaviors of male rats exposed to short-term and low-dose AgNPs increased, and those changes of exposure to AgNS were more pronounced.	[169]
BSA-coated AgNS: 20 ± 5 nm	Male rats: 10 weeks old	*ig*.: 1 and 30 mg/kg b.w./day, lasted for 28 days	- The memory and cognitive coordination processes of rats exposed to low dose AgNPs have been affected. - The presence of Ag^+^ in different brain regions, especially in the hippocampus, plays a crucial role in AgNPs induced impairment of advanced brain function.	[134]
CT-coated AgNPs: 10 ± 4 nm	Wistar rat pups: 2 weeks old	Oral gavage with a gastric probe: once daily at a dose of 0.2 mg/kg b.w./day for 21 consecutive days	- Silver concentrations in brain were 0.15 ± 0.01 mg/kg w.w. in AgNPs-treated group and 0.23 ± 0.03 mg/kg in Ag citrate-treated group measured by ICP-MS. - TEM analysis of the rat brains exposed to AgNPs revealed ultrastructural features indicative of the presence of ER stress. - The unfolded protein response (UPR) pathway mediates protective mechanisms in immature rat brains exposed to low doses AgNPs.	[170]
CT-coated AgNPs: 10 nm	Male mice: 4–5 weeks old	*ig*.: 0.25 and 1 mg/kg b.w./day AgNPs, silver acetate (AgAc) at a dose of 1.55 mg/kg b.w./day, once a day for 5 days per week, lasted for 4 weeks	- Abnormal behaviors and significant difference in body/organ weight were not observed. - The content of sliver accumulation in the brain tissue in AgAc- and AgNPs-exposed groups were dose-dependent at the end of treatment (EoT), ant, it was a significant difference in mice treated with AgAc and AgNPs compared with the control group treated with sterile water. After the recovery period, the total sliver in brain of mice treated with 0.25 mg/kg b.w. AgNPs, 1 mg/kg b.w. AgNPs and 1.55 mg/kg b.w. AgAc were reduced by 31%, 38%, and 50%, respectively. - The immunoreactive GFAP^+^ expression of astrocytes was significantly increased in the hippocampus of mice treated with AgNPs at the EoT, while there were no differences that could be observed after recovery. - The immunoreactive Iba1^+^ expression of microglial cells was significantly increased in the cortex of mice treated with 1 mg/kg b.w./day AgNPs at the EoT, and it significant decreased in all treated groups after recovery. - The split of the basement membrane of capillaries and the swelling of astrocytic perivascular endfeet were observed in the hippocampus of mice treated with 1 mg/kg b.w. AgNPs- and AgAc at the EoT, while no changes were found in the cortex. - The impact on glial cells and ultrastructural changes of the BBB caused by AgNPs affected the accumulation and slow clearance of silver in the brain.	[171]
AgNPs: 20–25 nm	Neonatal SD rats	intranasal instillation: 0.1, 0.2, 0.5, and 1 mg/kg b.w./day, once a day for 14 consecutive weeks	- Significantly decreased body weight. - The GFAP protein expression of cerebellar tissue in rats treated with AgNPs were dramatically increased, while it is significantly reduced in rats treated with AgNPs-Vitamin E compared with rats treated with AgNPs alone - The levels of activated caspase-3 in cerebella of rats treated with AgNPs significantly increased. - AgNPs exposure cause neurotoxic injury to the rat cerebellum by activating neuroglial cells and destroying the cerebellum granular layer, Vitamin E supplementation attenuates AgNPs-induced those neurotoxic injuries.	[27]
CT-coated AgNPs: 10 ± 4 nm	Rats: 2 weeks old, 30–40 g	*ig*.: 0.2 mg/kg b.w./ day, lasted for 21 days from postnatal day 14 (PND14)	- Silver content in brain were 0.15 ± 0.01 mg/kg w.w. in PND35 group and 0.17 ± 0.03 mg/kg w.w. in PND64 group measure by ICP-MS. - Behaviors of AgNPs-exposed rats were changed, AgNPs-exposed rats of PND14 group in a short time have pro-depressive and anti-anxiety reactions, but not in PND64 group.	[81]
AgNPs:	Adults male Wistar rats, average weight: 152 ± 20 g	Oral: 0.5, 5 and 10 mg/kg b.w./day, once a day for 14 consecutive weeks	- Minimal oxidative stress in the cortex and hippocampus in rat brains. - The reduction of AchE activity and the level of 5-HT and NE in rat brains were inhibited by AgNPs in a dose-dependent manner. - Brain tissues of rats treated with a dose of 0.5 mg/kg b.w. showed slight structural changes in the myelinated axons and their cytoplasm, and 5 mg/kg b.w. showed higher concentrations of AgNPs in the myelin lamella and their increased structural disturbance.	[99]
Uncoated AgNPs and PVP-coated AgNPs	Pregnant rats: 3 months old, Male offspring: 5 weeks after birth, the 1st day after copulation as the Gestation day 0 (GD0)	*i.p*.: 20 mg AgNPs/ml, 1 ml each time, once time every two days from GD10 to GD18	- The silver content in hippocampus of offsprings of pregnant rats exposed to uncoated AgNPs group (17.51 μg/g) were significantly higher than exposed to PVP-coated AgNPs (4.13 μg/g) and silver nitrate (5.31 μg/g). - The levels of GAP-43 protein expression in the hippocampus of offsprings brain of pregnant rats exposed to uncoated AgNPs were significantly lower than those exposed to PVP-coated AgNPs and silver nitrate, but there was no significant difference in the later two groups. - Pregnant rats exposed to uncoated AgNPs impaired spatial learning and memory ability in their offspring, while exposed to PVP-coated AgNPs protected from the toxic injury of their offspring.	[106]
AgNPs: 20 nm	Neonatal SD rats	Intranasal instillation: 0.1, 0.2, 0.5 and 1 mg/kg b.w./day, once a day for 14 consecutive weeks	- The protein and the mRNA levels of CACNA1A in CGCs decrease in a dose-dependent manner. - Cerebellar ataxia like symptoms (Dysfunction of motor coordination and impairment of locomotor activity) occurred in rats associated with CACNA1A expression decrease for the AgNPs-induced neurotoxicity. - The level of silver in cerebellum tissue increased from 12.2 ± 3.8 to 32.8 ± 5.2 μg/g by dose-related accumulation.	[120]
CT-coated AgNPs: 32 ± 6.6 nm	Female mice: 6–7 weeks old, Adult offsprings	*s.c*.: 0, 0.2 and 2 mg/kg b.w./day, female mice repeated administration once every 3 days from the 3rd day of gestation until parturition	- Cognitive behaviors in the Morris water maze of adult offspring significantly impaired; - Pregnant mice exposed to AgNPs show the number of defecations and leanings in the open field assay and number of passages in the light-dark box were greater in their offspring, Most of neurotoxic impairments were more apparent in their offspring which had been prenatally exposed to high-dose of AgNPs, especially female ones. - Pregnant animals exposed to AgNPs may cause neurobehavioral disorders in their offspring.	[172]
CT-coated AgNPs 9–21 nm, SN-coated AgNPs: 11–32 nm	Pregnant rats, Male offsprings: 6 weeks old	Oral: administration from the 10th day to the 21st day of gestation	- Pregnant rats exposed to AgNPs affected behaviors, and increased anxiety and higher levels of nitro-oxidative stress in their male offsprings. - The levels of nitro-oxidative stress and apoptosis in the hippocampus of CT-coated AgNPs group were higher than SN-coated AgNPs group, indicating functionalizing AgNPs with natural antioxidants can improve its stability and biocompatibility, and reduce toxic injury to CNS.	[173]
CT-coated AgNPs: 10 nm	Wistar rat pups: 2 weeks old	*ig*.: 0.2 mg/kg b.w./ day, Starting administration to pups at postnatal day 14 (PND14) and lasted for 21 consecutive days	- The concentrations of silver accumulated in brain tissue in AgNPs-exposed group were significantly higher than CT-coated AgNPs and saline exposure group at PND35 and PND90, and it below the detection limit value (0.011 mg/kg w.w.) in control group exposed to saline. - AgNPs and Ag^+^ accumulated in brains of immature rats affected the ultrastructure of the synapse. - Down-regulated the expression level of NMDA receptor complex-related proteins (GluN1 and GluN2B subunits, PSD95 and SynGAP). - The down-regulation of the GluN2B-PSD95-nNOS-cGMP signaling pathway, which maintains the process of LTP/LTD during development as a result of learning and memory formation, was the mechanism of the changes in NMDA receptors mediated by AgNPs. - The downregulation and density reduction of NMDA receptors and the disturbed molecular pattern of synaptic plasticity caused by disintegration of glutamatergic synapses were related to neurotoxicity in brain of immature rats exposed to Ag^+^ or AgNPs.	[174]
AgNPs: 18 ± 1.8 nm	Female mice: 4–5 weeks old, Offspring	Oral: 3 mg/kg b.w./day, Administration from 1 week before the mating of female and male mice to the end of parturition	- Increased likelihood of engaging in certain repetitive behaviors. - Decreased resident microglial cells in brain of offspring. - Increased body fat in the offspring.	[175]

*AgNS* Sphere-shaped AgNPs, *AgNC* Cube-shaped AgNPs, *BSA* Bovine serum albumin, *i.v*. intravenous, *i.p*. intraperitoneal, *s.c*. subcutaneous, *b.w.* body weight

## Potential neurotoxic injuries of dental restorative materials added with AgNPs

AgNPs have been widely added to restorative materials, including composite resins, adhesive materials, implant dentures, and denture bases, which are used in clinical prosthodontic treatment of endodontics, restorative dentistry, dental prosthesis, implantology, orthodontics, and periodontics [126]. In an oral microenvironment, dental restorative materials added with AgNPs are affected by saliva, chewing, and other factors. The continuously released AgNPs and their released Ag^+^ can enter the blood circulation by penetrating the oral mucosa or being absorbed in the digestive tract after they are swallowed; then, they are distributed to tissues and organs of organisms. The efficiency of AgNPs entering the blood circulation through the oral mucosa may be higher than that of oral administration [127, 128]. Interestingly, some studies have found that AgNPs and their released Ag^+^ can penetrate the oral mucosa. However, no significant difference is observed between the oral permeation fluxes of AgNPs and Ag^+^ (prepared by filtering AgNP solution), indicating that AgNPs are absorbed in the oral cavity through the released Ag^+^ [129]. The neurotoxicity of AgNPs has been confirmed by a large number of studies. Even exposure to low-dose AgNPs produces mild oxidative stress in brain tissues and causes neurotoxic reactions [130]. Therefore, although a low-dose proportion of AgNPs is added to dental restorative materials, AgNPs are continuously released by these materials in the mouth for a long time and can be absorbed into the blood circulation through the oral mucosa and gastrointestinal tract. Consequently, they accumulate in brain tissues, eventually causing neurotoxicity. Different from studies on the neurotoxicity of animal models exposed to AgNPs, few studies have been performed on the neurotoxicity caused by dental restorative materials added with AgNPs, we think that it may be limited to the low content of AgNPs added to dental restorative materials, resulting in too slow release rate of AgNPs, even if neurotoxicity occurs, it need to take a long time. Therefore, we have summarized neurotoxic studies of animal models exposed to AgNPs and then assessed the possibility of neurotoxicity caused by dental restorative materials added with AgNPs. These studies confirmed that the neurotoxic injury induced by AgNPs mainly depends on the physical properties and exposure dose of AgNPs, animal models (e.g., animal species, weight, gender and age) and administration time/route. Among those factors, the dosage and administration time of AgNPs exposed to animal models were the most critical factors. Similarly, we believe that whether dental restorative materials added with AgNPs caused neurotoxicity in models mainly depends on the dosage of AgNPs released from dental restorative materials and the exposure time. Owing to dental restorative materials need to remain in the patient’s oral cavity for a long time, we mainly consider the dosage of AgNPs released from those materials, that is, the release rate of AgNPs which depend on the proportion of AgNPs added in dental restorative materials. In vitro studies, it has confirmed that the higher proportion of AgNPs added in dental restorative materials, the stronger antibacterial performance. However, too higher proportion resulting poor physical/mechanical properties and biological safety of those materials. Therefore, we need to select the most appropriate addition proportion of AgNPs for dental restorative materials preparation. Finally, we think that once AgNPs released from dental restorative materials added with the appropriate addition proportion of AgNPs after long-term used in animal models reached the lowest dose of neurotoxicity, neurotoxic injury to CNS occurred. The release mode of AgNPs from dental restorative materials is’t remaining constant, it gradually decreases after burst release in the early phase and tends to be stable in the later phase. Rafał et al. [131] prepared Ti disks (Disc shape, 10 mm in diameter and 2 mm in thickness) modified their surface with AgNPs showed that the largest amount of Ag^+^ are released initially from the surface of the samples, and the burst released after 6 h immersion. Then, the amount of Ag^+^ gradually decreased and finally reached a stable value after 24 h of immersion. Radtke et al. [132] doped AgNPs on the surface of species (7 × 7 mm, 0.20 mm in thickness) prepared with Ti6Al4V (titanium alloy) and Ti6Al4V/TNT5 (Nanotubular modified titanium) and immersed in the PBS solution, results of Ag^+^ concentration released from those samples showed that samples with enriching 2.3 wt% AgNPs surface was rapidly increased in the first 10 days and then remained at the level of 1.7~2 ppm, Ti6Al4V samples with enriching 1.1 wt% AgNPs surface reached the maximum Ag^+^ release after 7 days and remains at the level of 0.9~1.1 ppm, Ti6Al4V/TNT5 samples with enriching 1.0 wt% AgNPs was lower than 0.1 ppm in the first 10 days and reached the highest concentration 0.4 ppm after 34 days. In the research of PMMA, Nam et al. [133] prepared disc specimens (20 mm in diameter and 3.0 mm in thickness) by denture base acrylic incorporated with AgNPs at the concentrations of 1.0, 5.0, 10.0, 20.0 and 30.0 wt%, they found that the Ag^+^ concentration released from specimens were increased from 0.176 mg/l to 0.356 mg/l at 24 h, and 0.119 mg/l to 0.268 mg/l at 30 days. Most important of all, Animal models exposed to AgNPs (ig., 10 nm in diameter) at 0.2 and 0.25 mg/kg b.w./day for 14 days showed the expression of proteins and mRNA related to oxidative stress and myelin sheath in the brain were change, while there were no changes in behaviors and memory of models occurred [109, 130]. After 28 days of continuous exposure to AgNPs (ig., 20 nm in diameter) at 1 mg/kg b.w./day, the hippocampal region of the animal model was damaged and affected the memory and cognitive coordination processes of those models [134]. In addition, the release rate of AgNPs and Ag^+^ in dental restoration materials was related to the amount of materials used in the oral cavity. For example, the release dose of AgNPs will multiply increase after more implants used in prosthetic treatment. Therefore, once the accumulation of AgNPs and Ag^+^ in brain released from long-term use of dental restorative materials added with AgNPs reached the lowest neurotoxic level, toxic injury to the CNS occurred, but it still needs to be explored.

Although adding AgNPs alone can improve the antibacterial properties of dental restoration materials, but it also brings some problems, such as insufficient physical and mechanical properties and biocompatibility. Thus, AgNPs and nanomaterials with antibacterial or remineralization properties are used to prepare NP composite agents, which are added to dental restorative materials to develop new multifunctional dental restorative materials with enhanced remineralization properties, antibacterial effects, and physical/mechanical properties. More importantly, the release rate and mode of AgNPs contained in NP composite agent added in dental restorative materials have changed, causing the release dose of AgNPs decrease [135, 136]. Taking such an approach can reduce the incorporation ratio and release dose of AgNPs from dental restorative materials, and ultimately reduce or even eliminate the possibility of neurotoxic injury to CNS, except for the modification of the physical and mechanical properties of AgNPs. Unfortunately, nanomaterials such as metal oxides with antibacterial properties and nanoadditivess with remineralization properties may also induce neurotoxicity [137–140]. Similar to studies on the neurotoxicity of dental restorative materials added with AgNPs alone, no studies have been performed on neurotoxicity caused by the simultaneous incorporation of AgNPs and metal oxides, antibacterial agents, remineralizers, and other nanoparticles into dental restorative materials. and those processes may take several years or even decades. As such, scientific experimental models and evaluation methods for studying the neurotoxicity of dental restorative materials are difficult to establish. Therefore, these materials should be further explored.

## Conclusion and future perspectives

Neurotoxicity caused by low-dose AgNPs has been confirmed, especially in special populations, such as fetuses, infants, children, and the elderly. As such, implant and denture base materials remaining in the oral cavity of patients for a long time should be carefully monitored, especially during implant repair treatment. The surface coating of an implant is directly in contact with blood, so it easily causes the accumulation of nano additives in brain tissues. After entering the blood circulation in a short time, these additives become transported across the BBB. Composite resin materials and denture base materials are constantly worn and dissolved during use to release nanomaterials, which enter the blood circulation by penetrating the oral mucosa or being absorbed in the digestive tract after swallowing [139]. Therefore, the application of AgNPs in implant restoration and denture restoration should be more prudent, especially for special populations. However, few studies have been performed on the neurotoxicity caused by dental restorative materials added with AgNPs. Current experimental methods and models have remarkable limitations, resulting in difficulty in establishing scientific methods for evaluating the neurotoxicity of dental restorative materials added with AgNPs. Therefore, further scientific and reasonable research methods should be developed to evaluate the neurotoxicity and specific mechanism of dental restorative materials added with AgNPs and provide a theoretical basis for designing better.

## Abbreviations

AD: Alzheimer’s disease
AgNPs: Silver nanoparticles
Ag_2_S: Silver sulfide
ASC: Apoptosis associated speck like adapter protein
ALC: Acetyl L-carnitine
BBB: Blood-brain barrier
CaP: Calcium phosphate
CIP: Ciprofloxacin
CNS: Central nervous system
Cyt-c: Cytochrome-c
CT: Citrate
ECs: Endothelial cells
DNT: Double-layer TiO2 nanotubes
NSC: Nano-porous Silica
QADM: Quaternary ammonium dimethacrylate
GIC: Glass ionomer cements
ER: Endoplasmic reticulum
GSDM: Gasdermin
GFAP: Glial fibrillary acidic protein
GPx: Gglutathione peroxidase
H_2_S: Hydrogen sulfide
HA: Hydroxyapatite
ICAM-1: Intercellular adhesion molecule-1
ICP-MS: Inductively coupled plasma mass spectrometry
IFN-γ: Interferon-γ
LDH: Lactic dehydrogenase
LPS: Lipopolysaccharide
MCP-1: Monocyte chemokine-1
MDA: Lipid peroxidation
NLRP-3: NOD-like Receptor (NLR) family pyrin domain containing-3
NAC: N-acetylcysteine
NACP: Amorphouscalcium phosphate
NF-κB: Nuclear transcription factor-κB
NE: norepinephrine
PDA: Polydopamine
PDGFβR: Platelet-derived growth factor receptor-β
PSD-95: Postsynaptic receptor density protein95
PGE2: Prostaglandin E2
PMMA: polymethyl methacrylate
PVP: Polyvinylpyrrolidone
ROS: Reactive oxygen species
SiO_2_: Silicon dioxide
TEM: Transmission electron microscopy
TFEB: Transcription factor EB
TGF-ß: Transforming growth factor-ß
Ti: Titanium
TiO_2_: Titanium dioxide
TJs: Tight junctions
TNF-α: Tumor necrosis factor-α
YO-NPs: Yttrium Oxide nanoparticles
ZrO_2_: Zirconium dioxide
ZnO: Zinc oxide
ZO-1: Zonula occludens -1
3-MP: 3-methyladenine
-SH: Sulfhydryl
5-HT: 5-Hydroxytryptamine.
